# *Ehrlichia chaffeensis* Infection in the Reservoir Host (White-Tailed Deer) and in an Incidental Host (Dog) Is Impacted by Its Prior Growth in Macrophage and Tick Cell Environments

**DOI:** 10.1371/journal.pone.0109056

**Published:** 2014-10-10

**Authors:** Arathy D. S. Nair, Chuanmin Cheng, Deborah C. Jaworski, Lloyd H. Willard, Michael W. Sanderson, Roman R. Ganta

**Affiliations:** 1 Department of Diagnostic Medicine/Pathobiology, College of Veterinary Medicine, Kansas State University, Manhattan, Kansas, United States of America; 2 Department of Entomology and Plant Pathology, Oklahoma State University, Noble Research Center, Stillwater, Oklahoma, United States of America; University of Texas Medical Branch, United States of America

## Abstract

*Ehrlichia chaffeensis*, transmitted from *Amblyomma americanum* ticks, causes human monocytic ehrlichiosis. It also infects white-tailed deer, dogs and several other vertebrates. Deer are its reservoir hosts, while humans and dogs are incidental hosts. *E. chaffeensis* protein expression is influenced by its growth in macrophages and tick cells. We report here infection progression in deer or dogs infected intravenously with macrophage- or tick cell-grown *E. chaffeensis* or by tick transmission in deer. Deer and dogs developed mild fever and persistent rickettsemia; the infection was detected more frequently in the blood of infected animals with macrophage inoculum compared to tick cell inoculum or tick transmission. Tick cell inoculum and tick transmission caused a drop in tick infection acquisition rates compared to infection rates in ticks fed on deer receiving macrophage inoculum. Independent of deer or dogs, IgG antibody response was higher in animals receiving macrophage inoculum against macrophage-derived *Ehrlichia* antigens, while it was significantly lower in the same animals against tick cell-derived *Ehrlichia* antigens. Deer infected with tick cell inoculum and tick transmission caused a higher antibody response to tick cell cultured bacterial antigens compared to the antibody response for macrophage cultured antigens for the same animals. The data demonstrate that the host cell-specific *E. chaffeensis* protein expression influences rickettsemia in a host and its acquisition by ticks. The data also reveal that tick cell-derived inoculum is similar to tick transmission with reduced rickettsemia, IgG response and tick acquisition of *E. chaffeensis*.

## Introduction

*Ehrlichia chaffeensis* is, an obligate, intracellular, Gram negative bacterium belonging to the family Anaplasmataceae. It is transmitted by the bite of an infected tick, *Amblyomma americanum* (lone star tick) [1], [2], and is responsible for an emerging disease, human monocytic ehrlichiosis (HME) [3]–[5]. The symptoms of HME are variable and may include fever, myalgia and headaches [6]–[8]. Severe and potentially fatal outcomes are documented in elderly and immunocompromised individuals [6], [7]. *E. chaffeensis* also infects several other vertebrate hosts, such as dogs, goats, coyotes and white-tailed deer [2], [9]–[13].

White-tailed deer is identified as the reservoir host of *E. chaffeensis* [2], while humans, dogs and other vertebrate animals are considered incidental hosts [14]. Research on *E. chaffeensis*, focused on understanding the host response, has been carried out mostly in mice or *in* *vitro* using infection inoculum originating from canine or human macrophage/monocyte cell lines [15]–[23]. Mouse is not a natural host for acquiring infection from a tick and moreover infections in this host are cleared fairly rapidly (within about 14 days), particularly with the inoculum originated from vertebrate macrophages [15], [18]–[20], [24]. Several recent studies reported numerous differences in the transcriptome and proteome of *E. chaffeensis* originating from macrophage and tick cell cultures [25]–[28]. We reported earlier that mice infected with tick cell culture-derived and macrophage culture-derived *E. chaffeensis* vary in clearing the pathogen and in inducing immune response [20]. These studies suggest that the pathogen progression in a host depends on the source of the inoculum and that the most natural inoculum possible is needed to allow for a realistic understanding of the pathogenesis caused by *E. chaffeensis* in a vertebrate host. Further, we hypothesized that understanding the pathogenesis and immunity requires infection assessment in hosts where *E. chaffeensis* infections occur naturally.

In this study, we compared infections in deer with intravenous (i.v.) inoculation with macrophage and tick cell cultured organisms as well as by tick transmission. In addition, we carried out infections in dogs and compared the infection progression in the reservoir and incidental hosts, white-tailed deer and dog, respectively. The data presented in this study demonstrate that tick cell-derived *E. chaffeensis* infection inoculum is the closest to tick transmission.

## Materials and Methods

### *In vitro* cultivation of *E. chaffeensis*

*E. chaffeensis* (Arkansas isolate) was continuously cultivated in the canine macrophage cell line (DH82) essentially as described earlier [29]. It was also cultivated in ISE6 tick cell line originated from *Ixodes scapularis* as in [25], [30]. Detailed protocols for propagating the organisms were followed as described earlier [31].

### Animals

One to three day-old white-tailed deer fawns, purchased from a breeder, were reared in a tick-free environment until the age of 3–5 months prior to performing experimental infections as described earlier [32]. Deer rearing and experimental infections were performed at the Oklahoma State University (OSU) and as per the approved protocol by the OSU Institutional Animal Care and Use Committee (IACUC). For dog infection experiments, six eight-month old specific pathogen free male beagles, purchased from a USDA approved vendor (Covance Research Products, Denver, PA) and housed in a climate controlled animal facility of Kansas State University (KSU), were used. All dog infection experiments were performed as per the approved protocol by the KSU IACUC.

### Animal infections

*E. chaffeensis* cultures in T150 flask growing in DH82 or ISE6 cell line were harvested at about 80–90% infectivity, centrifuged at 15,000×g for 10 min at 4°C, supernatant was discarded and the culture pellet was suspended in 15 ml of 1x phosphate buffered saline (PBS). The washing steps were repeated twice and the final cell pellet was resuspended in PBS to concentrate infected DH82 cells to about 2×10^6^ per ml (estimated concentration of *Ehrlichia* organisms was approximately 2×10^8^ per ml). One ml each of the cell suspension was used for intravenous (i.v.) injections per animal (deer or dog). The experiments in deer were performed two independent times with freshly prepared DH82 culture-derived inocula (two deer per infection) or once using the ISE6 culture-derived inocula (three deer). Two deer were kept as uninfected controls. Dogs were infected with *E. chaffeensis* infected DH82 or ISE6 cultures (two per inoculum). Two dogs were also kept as uninfected controls. Uninfected controls did not receive any inoculum as prior studies revealed no serological responses against host proteins when uninfected cells were used as the inoculum in mice or deer [11], [18]–[20], [33]. Both infected and control group animals were monitored for several weeks post infection for various parameters; clinical signs (changes in body temperature and behavioral changes), hematological changes, presence of rickettsemia, antibody responses and infection acquisition by ticks (details provided below). At the end of the study, all animals were euthanized and tissue samples were collected for further analysis. Infection experiments by tick transmission were also carried out in four deer. The details of tick attachment for the pathogen transmission are described below.

### Blood analysis

About 3 ml each of whole blood in EDTA tubes was collected aseptically at different post infection days starting from day three (once or twice a week) and used for assessing the hematological changes, infection status and for the pathogen-specific IgG responses. The blood samples up to 14 days were also used to prepare smears on glass slides, stained with Wright’s stain and examined for the presence of *E. chaffeensis* inclusions in the mononuclear cells. Typically, each slide was examined up to 20 fields under an oil immersion objective (1000 × magnification). Complete blood counts were performed at the clinical pathology laboratories of KSU or by Antech Diagnostics, Irvine, CA (via OSU). The hematological values were compared with the average range of values observed on day 0 of infected animals and with those observed for uninfected controls.

### Evaluation of blood samples for infection by culture and PCR

The infection progression in deer and dogs was monitored by culture isolation and by semi-nested polymerase chain reaction (PCR). Genomic DNA isolation and PCR assays were carried out as described recently [34], except that the PCR was targeted to Ech_1143 (p28-Omp 19) gene segment of *E. chaffeensis* Arkansas isolate. For the first round PCR, the primer pairs used were RRG34 (5′ GAAGCGCAATATCCAACTCCTC) and RRG18 (5′AACTAATAATTACAATGTGTG). For the second PCR, 2 µl of the PCR product from the first reaction was used as the template with primers RRG 34 and RRG 77 (5′CTACTCATGTCTGCTGCTGAG). PCR products (10 µl) were resolved by electrophoresis in 1.5% agarose gels containing ethidium bromide [35]. Positive samples were identified by comparing with the predicted PCR product obtained for a reaction containing *in* *vitro* culture-derived *E. chaffeensis* genomic DNA as the template.

### Acquisition and transmission feeding of ticks and infection monitoring

Nymphal *A. americanum* ticks were obtained from the National Tick Research and Education Resource (Tick Facility) at OSU. Ticks were propagated as in [36] and in accordance with the approval from the OSU IACUC. About 200 nymphal ticks were placed on day 5 post i.v. infections on deer. Similarly, ticks were allowed to feed on control animals. Engorged nymphs, dropped after complete blood meals (typically took about 5–7 days), were collected and allowed to molt to adults by incubating at room temperature in a 96% humidity chamber. Genomic DNA was isolated from randomly selected individual adult ticks using the Wizard SV Genomic DNA purification kit as per the protocol for DNA isolation from a tissue sample (Promega, Madison, WI), except that the isolated DNA was solubilized in 50 µl of rehydrartion buffer. Infection rates in the molted ticks were assessed by PCR (as above); 2 µl each of the DNA was used for PCR analysis.

Ticks having the highest infection rates were used for tick transmission experiments to naïve white tailed deer (originated from deer i.v. infected with DH82 culture inoculum). For pathogen transmission experiments, adult ticks (34 males and 20 females on Deer 10, 33 males and 21 females on Deer 11, 72 females and 69 males on Deer 12, 64 females and 26 males on Deer 13) were placed into a tick feeding chamber attached on each deer and allowed to feed for 7 days. Animal monitoring for infection and the pathogen-specific IgG expression was carried out as described above. Forty adult unfed pathogen-free ticks each (20 each of males and females) were also allowed to feed for 7 days on animals in the group on day 56 post transmission feeding. Genomic DNA recovered from these ticks was assessed for the infection rates by PCR (as above).

### Transmission Electron Microscopy (TEM)

The TEM of the tissue specimens were conducted [37] with the following tick-specific modifications to the protocol. Presumed infected ticks were dissected longitudinally and half tick each was transferred to ice cold 0.1 M cacodylate buffer (pH 7.4) containing 2.5% gluteraldehyde and were used for processing for TEM analysis. The second halves of the ticks were transferred to a tube containing 100% ethanol and subsequently used to isolate total genomic DNA and to perform PCRs (as above) to define the *E. chaffeensis* infection status. The ticks tested positive for *E. chaffeensis* were then used for the TEM analysis. Tick tissue sections stained with uranyl actetate and lead citrate were examined under a Hitachi H-300 electron microscope (Hitachi High-Tech, San Jose, CA) for the presence of *Ehrlichia* inclusions. Images were captured and developed by including a photograph scale marker as in [37]. For infection comparison, the TEM images generated for *E. chaffeensis* infected ISE6 tick cell culture were used [37].

### Enzyme linked immunosorbant assay (ELISA) to monitor *E. chaffeensis*-specific IgG expression

Host cell free *E. chaffeensis* lysates were prepared from the organisms recovered from infected DH82 and ISE6 cultures [38] and the lysates were used for the ELISA analysis [18]. Briefly, host cell free *Ehrlichia* antigens were prepared by following the protocol involving sonication of 80–90% infected culture, low speed centrifugation at 400×g for 5 min to remove nuclei and unbroken host cells, filtration of the supernatant through a 2.7 µm filter followed by a 1.6 µm filter to recover the host cell-free *Ehrlichia* organisms, then the organisms were pelleted by high speed centrifugation at 15,557×g, and finally washed three times with SPK buffer to remove any residual host cell antigens. The final purified organisms were used for preparing *E. chaffeensis* antigens for coating the ELISA plates. We also performed the ELISA analysis with uninfected DH82 and ISE6 cell antigens to check for the IgG responses against the host cells. Plasma samples from deer and dogs used in infection experiments and from control animals were used for the ELISA analysis to detect *E. chaffeensis*-specific antibody responses and against host cell proteins. Briefly, 96-well plates were coated with cell-free *E. chaffeensis* antigens or host cell antigens (20 ng/well). Plasma samples diluted to 1∶50 were used as primary antibody. Horseradish Peroxidase (HRP) conjugated anti-deer IgG antibody (KPL Inc., Gaithersburg, MD, USA) at a dilution of 1∶5000 was used as the secondary antibody. For dog samples, the ELISA was done using canine anti IgG ELISA kit (Bethyl Laboratories Inc., Montgomery, TX, USA). The secondary antibody (also HRP conjugated) dilution used in this experiment was 1∶50,000. The absorbance was measured at 450 nm using an ELISA plate reader (Spectramax, Molecular Devices, CA, US) to provide a quantitative optical density (OD). All assays were performed in triplicate wells and the average values were used. The data are presented as the change in OD calculated by subtracting the OD values of day zero samples from those for samples collected on subsequent days.

### Evaluation of tissue samples from infected deer and dogs for the presence of *E. chaffeensis*

At necropsy, spleen and liver tissues were collected. Total genomic DNA was isolated from about 20 mg each of tissue samples using Wizard SV Genomic DNA purification kit as per the manufacturer’s instructions (Promega, Madison, WI) and the final DNA was eluted into 200 µl of TE buffer. Two microliters of the eluted DNA was used for nested PCR analysis. The tissue samples from dogs were also similarly assessed by PCR and for pathological changes.

### Statistical analysis

The quantitative ELISA response in animals to DH82 or ISE6 cell culture-derived *E. chaffeensis* antigens was assessed; DH82 culture-derived *E. chaffeensis* infected deer (D), ISE6 culture-derived *E. chaffeensis* infected deer (I) and tick transmitted deer (T) were analyzed using the software, Stata 12.1 (Stata Corp LP, College Station, TX). To maximize power to detect differences an analysis of variance accounting for the repeated measures on animals over time and the nesting of animal within each antigen group was performed. Similar analysis was also carried out for dogs infected with DH82 and ISE6 culture-derived *E. chaffeensis* inocula.

## Results

### Evaluation of *E. chaffeensis* infection in deer infected with organisms cultured in macrophage or tick cell lines

To test the hypothesis that the origin of *E. chaffeensis* would impact the host response, we performed infection assessments over several weeks in deer infected i.v. with canine macrophage cell line (DH82) (n = 4) or with ISE6 tick cell line (n = 3) cultured *E. chaffeensis* inocula. The animals were assessed for the 1) clinical signs and hematology (performed only for animals infected with DH82-derived inoculum), 2) presence of the organism in blood assessed by PCR and culture recovery methods, 3) infection acquisition by ticks, and 4) IgG antibody responses.

***1) Clinical signs and hematology:*** An increase in body temperature by about 1.72°F was observed in all deer infected with DH82 derived inoculum compared to pre-infection body temperature of the same animals and that observed for uninfected controls (102.6°F±0.1) (Table 1). The mild fever was consistently observed on days when animals tested positive for the rickettsemia. Increased total leukocyte count was the only hematological abnormality observed as a result of infection. About 1.3 to 2.7 fold increases in the total leukocyte counts were observed following infection compared to the counts observed in the same animals prior to infection and in non-infection controls (Table 1).

**Table 1:** Clinical and hematological changes in deer infected with *E. chaffeensis*.

	Animalnumbers	Bodytemperature*(DPI)	Clinicalsigns	Total LeukocyteCount(TLC)/µl(DPI)	Foldchange inTLC
Needle infected animals					
DH82	1	104.3°F (21),104.0°F (35)	coughing, lethargic	1.7×103 (normal),2.2 to 2.5×103(5, 14)	1.3 to 1.5
	2	104.6°F (17),104.6°F (21),104.1°F (28)	none	no change	no change
	3	104.5°F (17),104.1°F (20)	none	2.7×103 (normal),4.4 to 5.9×103(3, 5, 12,17,21,28)	2.0 to 2.2
	4	104.7°F (0),104.0°F (5)	none	2.8×103 (normal),5.9 to 7.8×103(7,14)	2.0 to 2.7
Control	5	normal	none	not done	NA
	6	normal	none	no change	no change
Tick transmitted animals					
	10	104.1°F (17), 105.7°F (28), 104.7°F (32), 104.1°F (35), 104.5°F (42), 104.4°F (46)	none	2.1×103 (normal),3.3 to 4.0×103(18, 28 to 42)	1.6 to 1.9
	11	104.2°F (28)	coughing	2.1×103 (normal),4.1 to 5.5×103(18,28,32)	2.0 to 2.6

*Normal body temperature for the controls and for the animals prior to infection was 102.6±1°F.

***2) Detection of the organisms in blood:*** Deer blood was monitored once every three to four days (up to 41–63 days post infection) for the presence of viable organisms recovered by *in* *vitro* culture and by PCR analysis. Blood positives were detected more frequently (about 80% of the time; 35 of the 44 samples tested positive) in deer infected with DH82 cultured *E. chaffeensis* (Table 2). *E. chaffeensis* also persisted in deer infected with ISE6 cell-derived inoculum; the blood samples which tested positive for the pathogen in this group of animals, however, were considerably less and detected only about 28% of the time (10 of the 36 samples tested positive) (Table 3). The pathogen persistence was confirmed in both infected groups at the end point of the study when tissue samples were assessed (Tables 2 and 3).

**Table 2:** Culture recovery and PCR evaluation of the *E. chaffeensis* infection status in deer with DH82 culture derived inoculum.

Deer #	Days post infection																		Totalpositives/assessed(% positives)*
	0	3	7/8	10	13/14	17/18	20/21	23	28/30	35/37	49			52	59	63			
											blood	liver	spleen			blood	liver	spleen	
1	-	-	c	c	c	c	c	N	c	c	c			-	C	c	p	p	10/12 (83)
2	-	-	c	c	c	c	c	N	c	-	-			-	C	-	p	p	8/12(66)
3	-	p	p	c	c/p	c/p	-	c/p	c/p	p	c/p	p	p						9/10(90)
4	-	p	p	-	c/p	p	-	p	p	p	p	p	p						8/10 (80)
5	-	-	-	-	-	-	-	-	-	-	-	-	-						0/12 (0)
6	-	-	-		-	-	-	-	-	-	-	-	-						0/12 (0)

The letters c, p or c/p refer to any sample tested positive by culture recovery or PCR or both, respectively. The letter N represents the days on which blood samples were not collected.

*Total average positives in infected animals are 80% (day 0 data were not included in this calculation).

**Table 3:** Culture recovery and PCR evaluation of the *E. chaffeensis* infection status in deer with ISE6 culture derived inoculum.

Deer #	Days post infection															Total positives/assessed(% positives)*
	0	5	7	10	14	18	20	24	27	31	34	38	41			
													blood	liver	spleen	
7	-	c	-	-	c	-	-	-	-	-	c	c	-	-	p	5/12 (42)
8	-	-	-	-	-	-	-	-	-	-	c	-	-	p	-	2/12(17)
9	-	c	-	c	-	-	-	-	-	-	-	-	-	p	-	3/12(25)

The letters c, p or c/p refer to any sample tested positive by culture recovery or PCR or both, respectively. The letter N represents the days on which blood samples were not collected.

*Total average positives in infected animals are 28%.

***3) Infection acquisition by A. americanum ticks:*** On day 5 of post infection, about 200 unfed *A. americanum* nymphs per animal were allowed to feed to repletion on all infected animals and on uninfected controls. Following molting of the fed nymphs, adult ticks of both sexes randomly selected from every animal were assessed for the presence of infection by the PCR assay targeting to Ech_1143 gene. Ticks acquisition fed on deer infected with DH82-derived *E. chaffeensis* had an infection rate of 78% (38 out of 49 tested positive), while ticks fed on ISE6 tick cell-derived *E. chaffeensis* infected animals were positive only about 28% of the time; since our initial assessment of 50 ticks in this pool revealed low infection rate, we evaluated about three times more ticks to confirm the infection rate (45 of the 156 ticks tested positive). Ticks fed on uninfected animals were negative for the pathogen (20 ticks were tested).

**Figure 1:**
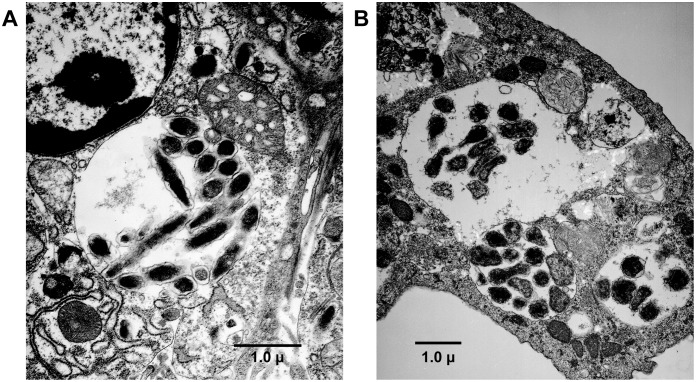
Transmission electron microscopy (TEM) analysis of *E. chaffeensis* infected ticks. *E. chaffeensis* organisms in a phagosome (morula) within the midgut cell of a PCR positive tick were observed under TEM (A). The organisms in the tick midgut tissue phagosome appear morphologically very similar to those observed in the *in* *vitro* cultured ISE6 tick cell phagosome (B) having a morula membrane, intra-morula space, inner and outer membranes and having inclusions of both dense core and elongated (dividing) cells.

Several ticks from both infected and uninfected animals were also assessed for the presence of *E. chaffeensis* inclusions by transmission electron microscopy. Intracellular vacuoles containing inclusions of *E. chaffeensis* organisms were difficult to see in ticks with the exception of one male PCR positive tick that was recovered from a DH82-derived inoculum infected deer; inclusions were observed in the gut epithelium of this tick (Figure 1A). The inclusions included both dense core and reticulate cells undergoing binary fission and they contained clearly distinct outer and inner membranes. The inclusions found in the tick were very similar to those observed in the ISE6 tick cell culture infected with the *E. chaffeensis* (Figure 1B) [37].

**Figure 2:**
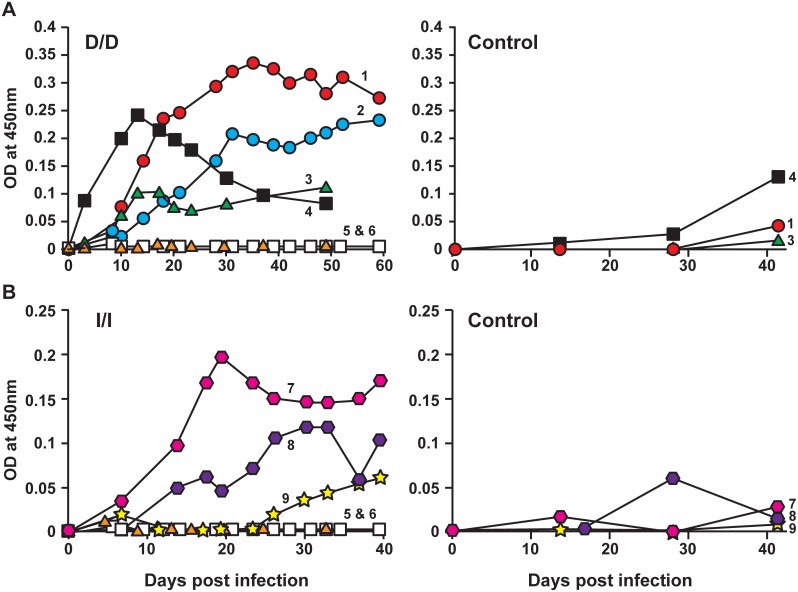
Antibody response of deer i.v. infected with *E. chaffeensis* culture in DH82 cells or in ISE6 cells (left panels of A and B, respectively). D/D represents both infection inoculum and *E. chaffeensis* antigens used for ELISA are from DH82 cultures, while I/I refers to both the infection inoculum and the ELISA antigens are obtained from ISE6 cultures. Deer 1–4 and 7–9 are infected animals and deer #s 5 and 6 are uninfected controls. The animals were also assessed for the IgG response against uninfected DH82 and ISE6 host cell antigens (right panels of A and B, respectively).

***4) IgG response against E. chaffeensis:*** IgG responses specific to DH82- and ISE6-derived *E. chaffeensis* antigens were evaluated by ELISA to determine if there were any variations in deer receiving macrophage- or tick cell-grown inocula. In this experiment, we purified host cell-free *E. chaffeensis* organisms from infected DH82 and ISE6 cultures and the pathogen antigens were then prepared and used to assess the IgG responses in animals infected with DH82 and ISE6 cell-derived inocula, respectively. *E. chaffeensis*-specific IgG antibody expression steadily increased and persisted in most of the infected animals (Figure 2A and 2B). Host cell antigens were also used to assess if there is any xenogeneic response against host cell antigens; consistent with the prior observations [11], [18]–[20], [33], the animals did not exhibit IgG response against host cell antigens (Figure 2A and 2B control panels).

### Evaluation of *E. chaffeensis* infection in deer by tick transmission

**Figure 3:**
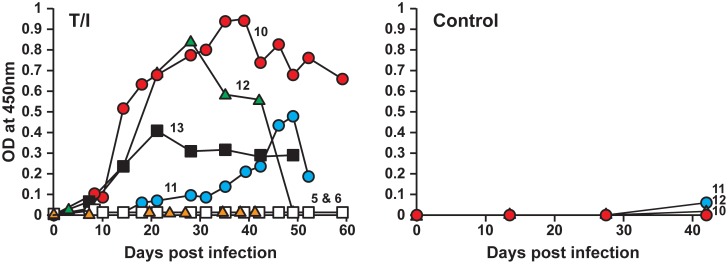
Antibody response of deer receiving infection by tick transmission. T/I refers to the animals receiving infection via tick bite and the *Ehrlichia* antigens used for the ELISA analysis are obtained from ISE6 cultures. Deer #s 5 and 6 are uninfected controls and deer #s 10–13 represent animals receiving infection by tick transmission. Control panel on the right refers to the ELISA analysis performed on sera from animals using the uninfected ISE6 host cell antigens.

Adult ticks having about 78% infection (generated in the previous experiment) were used to assess the pathogen’s transmission from ticks to deer. The primary goal of this experiment was to evaluate the impact of tick transmission on the pathogen’s progression and host IgG response relative to that observed in deer receiving i.v. infection of macrophage- and tick cell-derived inocula (described above). Clinical signs, hematological assessment and the presence of infection in blood were carried out as described above. As in the i.v. infected animals, all deer in this group (n = 4) also exhibited mild increases in body temperature after 10 days post infection (Table 1). Hematological changes were more pronounced in this group compared to i.v. infected animals. In one animal (deer 10), the changes included the drop in platelet count from the pre inoculation value of 5.86×10^5^/µl to 1.78×10^5^/µl and 2.47×10^5^/µl on days 28 and 42 post tick attachment, respectively. Leukocytosis was also observed in these animals on day 18 onwards which persisted up to 32 or 42 days. The leukocytosis paralleled with elevated body temperature for these animals (Table 1). The fold increase in the total leukocyte count was very similar to that observed in needle inoculated animals (1.6–2.6 fold increase) compared to pre-inoculation values (Table 1). Blood samples from all four deer tested positive for *E. chaffeensis* by culture recovery and by PCR (Table 4). The overall frequency of the pathogen detection in blood was about 27% (12 out of 44 samples tested). Infection rate in ticks allowed to acquisition feed on these animals was considerably lower at 8% (only three out of 38 ticks tested positive) than that observed for ticks fed on deer i.v. infected with DH82 culture-derived inoculum (78%) or with ISE6 culture-derived inoculum (28%) (described above). IgG response in all four animals steadily increased against *E. chaffeensis* starting from day 10 post tick attachment, although animal to animal variations were observed (Figure 3). The animals did not exhibit any IgG response against the host cell antigens (Control panel in Figure 3).

**Table 4:** Culture recovery and PCR evaluation of the *E. chaffeensis* infection status in deer infected by tick transmission.

	Days post infection																			Total positives/assessed (% positives)*
Deer #	0	7	10	14	18	21	24	28	32	35	39	42	46	49	52	59	63			
																	blood	liver	spleen	
10	-	-	-	p	-	-	-	p	-		-	-	-	-	-	-	-	-	-	2/15 (13)
11	-	c	c/p	p	-	-	-	p	-		-	-	-	-	-	-	p	-	p	5/15 (33)
12	-	-		-		c		c		-		-		-						2/7 (28)
13	-	c		c		-		-		-		p		-						3/7 (42)

The letters c, p or c/p refer to any sample tested positive by culture recovery or PCR or both, respectively. Blank cells represents the days on which samples were not collected.

*Total average positives are 27%.

### Differential antibody responses observed in deer for *E. chaffeensis* originating from tick cell environment versus macrophage cell environment

**Figure 4:**
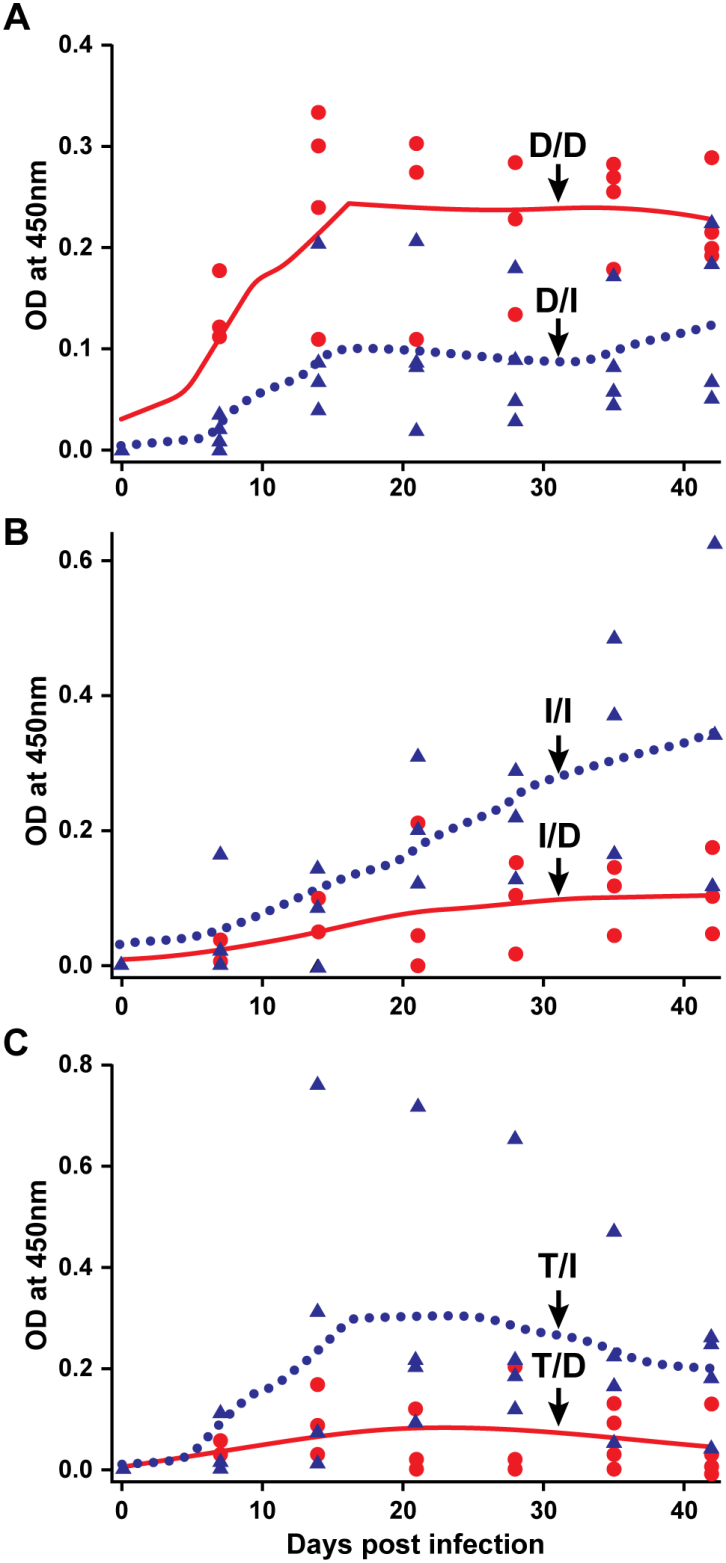
*E. chaffeensis*-specific IgG response in deer receiving infection by i.v. with DH82 culture-derived inoculum (panel A) or i.v. with ISE6 culture-derived organisms (panel B) or by tick transmission (panel C). Black circle data points and black line (polynomial smoothened line) in panel A refer to IgG data for DH82 culture infected animals using DH82 culture-derived antigens (D/D), while the black triangles and dotted line represent IgG response data generated with ISE6 culture-derived antigens (D/I). Panel B; as in A, except that the deer in this group were infected with ISE6 culture-derived inoculum and antigens used in the ELISA analysis are either from DH82 (I/D) (black circle data points and black line) or ISE6 (I/I) (black triangles and dotted line). Panel C represent IgG data for tick-transmitted animals and antigens used in the ELISA analysis are either from DH82 (T/D) (black circle data points and black line) or ISE6 (T/I) (black triangles and dotted line) culture derived. The Y-axis scales differ between charts.

We observed greater similarities in the pathogen’s persistence for the tick cell-grown *E. chaffeensis* and tick transmission (judged by the frequency of the pathogen detection in blood and by xenodiagnosis in ticks; described above); therefore, we hypothesized that the antibody response for these two groups of animals would be similar. To test this hypothesis, we compared the IgG responses against *E. chaffeensis* in all three groups of infected animals (i.e., the two i.v. infected groups and the tick transmitted group). The analysis was performed using the *E. chaffeensis* antigens originating from tick cell (I) and macrophage (D) cultures (Figure 4). Deer i.v. infected with macrophage culture-derived *E. chaffeensis* had significantly higher antibody response when assessed with the antigens prepared from macrophage cultured *E. chaffeensis* (D/D; homologous antigens) than with antigens prepared from tick cell-derived *E. chaffeensis* (D/I; heterologous antigens) (p values<0.01) (Figure 4A). Similarly, deer infected with tick cell-derived *E. chaffeensis* had significantly higher IgG response against homologous antigens (I/I; tick cell-derived bacterial antigens) compared to that observed for macrophage culture-grown bacterial antigens (I/D; heterologous antigens) (p values<0.01) (Figure 4B). IgG responses in deer receiving infection by tick transmission (the natural mode of transmission) was significantly higher to the tick cell-derived bacterial antigens (T/I) compared to that observed for macrophage culture-grown *E. chaffeensis* antigens (T/D) (p values<0.01) (Figure 4C). Specifically, the tick cell inoculum and tick transmission were not different (p>0.5) in IgG responses against tick cell-derived *E. chaffeensis* antigens.

### *E. chaffeensis* infection progression in dogs was similar to that observed in deer

Deer infection experiments (described above) revealed greater similarity in the pathogen persistence and IgG responses in deer receiving the tick cell-derived *E. chaffeensis* inoculum to that observed when the pathogen was transmitted by infected ticks compared to macrophage-derived inoculum infected animals. In parallel to these observations, we tested the hypothesis that the *E. chaffeensis* infection persistence and IgG responses in an incidental host would also be similarly specific to an inoculum. Experimental i.v. infections were performed in beagle dogs with *E. chaffeensis* grown in DH82 and ISE6 cultures. The infected dogs were assessed for 44 days for clinical signs, hematology, persistence of the pathogen, and for the IgG responses. The average body temperature of control animals and pre inoculation values of infected groups is 102°F±0.4. In the DH82 culture infected group, dog 7 exhibited an elevated body temperature of 103–104°F from day 9 onwards to the end of the study period. In ISE6 infected group, dog 4 also exhibited persistent fever (103.2–103.6°F) from day 9 post infection onwards. Dog 5 in this group also had fever (103–103.3°F) which also started on day 9 and persisted up to day 29 post infection. A drop in hemoglobin was also observed (12.5–12.9 g/dL) three of the four (dogs 4, 6 and 7) infected dogs between days 5–9 post infection. A moderate decline in total leukocyte count was noted (5.7–6.0×10^3^ cells/µL) in one dog (dog 5) infected with ISE6 culture inoculum on day 5 post infection. A reduced PCV (<38) was observed between days 5–16 post infection for three dogs (dogs 4, 6 and 7). A decline in platelet count was observed in dog 5 from day 29 onwards (1.06–1.52×10^5^) compared to control levels (1.64–5.10×10^5^ cells/µL).

**Figure 5:**
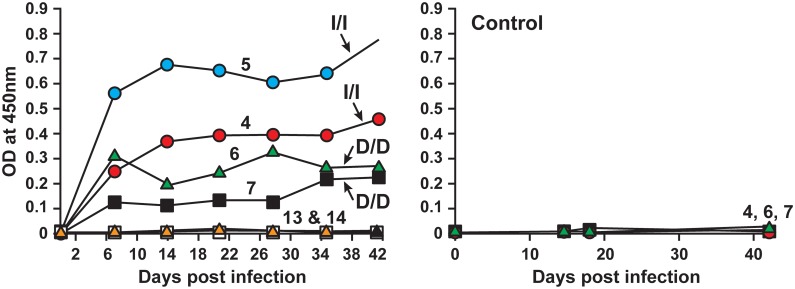
Antibody response in dogs i.v. infected with *E. chaffeensis* originating from ISE6 culture (dogs 4 and 5) and DH82 culture (dogs 6 and 7). D/D and I/I are as described in Figure 2. Dogs 13 and 14 are uninfected controls. Control panel on the right refers to the ELISA analysis performed on sera from animals using the uninfected host cell antigens.

**Figure 6:**
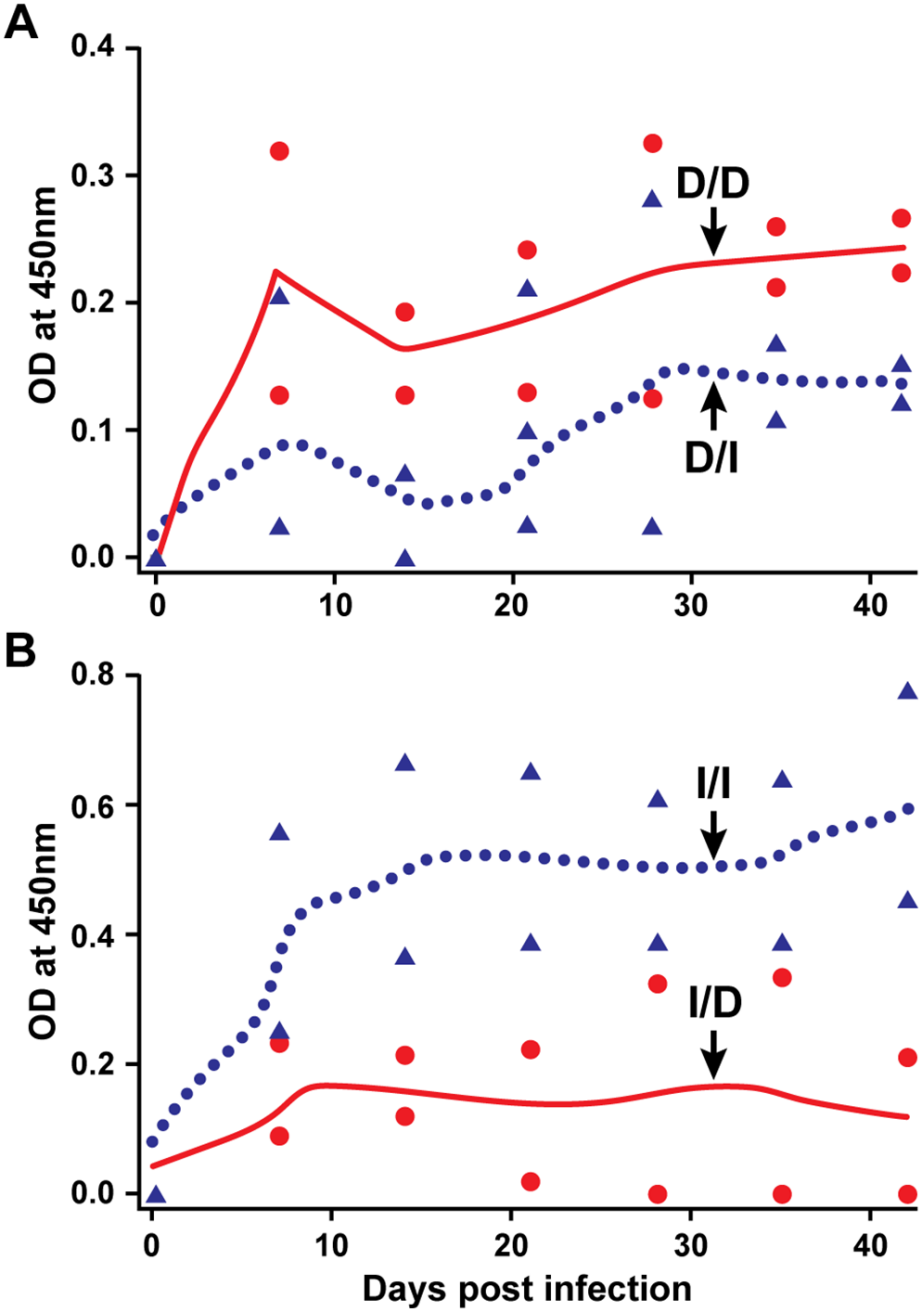
*E. chaffeensis*-specific IgG response in dogs receiving infection by i.v. with DH82 culture-derived inoculum (A) or i.v. with ISE6 culture-derived organisms (B). Black circle data points and black line (polynomial smoothened line) in panel A refer to IgG data for DH82 culture infected animals using DH82 culture-derived antigens (D/D), while the black triangles and dotted line represent IgG response data generated with ISE6 culture-derived antigens (D/I). Panel B; as in A, except that the dogs in this group were infected with ISE6 culture-derived inoculum and antigens used in the ELISA analysis are either from derived from DH82 (I/D) (black circle data points and black line) or ISE6 (I/I) (black triangles and dotted line) cells. The Y-axis scales differ between charts.

As in deer, higher frequency of the pathogen detection was observed in dogs infected with DH82 culture-derived inoculum (83% of the time; 20 of the 24 samples tested positive), whereas the detection frequency in dogs infected with ISE6 culture-derived inoculum was considerably lower (detected only at 33% of the time; 8 of the 24 samples tested positive) (Table 5). One tick cell inoculum infected animal tested negative by PCR and culture for all post infection days; the animal was positive for the infection, as the infection was confirmed in tissue samples at the end point of the study. All infected animals also exhibited a persistent *E. chaffeensis*-specific IgG response detectible from 7 days post infection (Figure 5). The IgG response was greater for the DH82 culture-derived antigens in dogs infected with DH82-derived inoculum (D/D; homologous antigens) compared to that observed for the same animals for ISE6 cell derived antigens (D/I; heterologous antigens) (p values<0.01) (Figure 6). Similarly, dogs infected with ISE6 culture-derived inoculum had higher IgG response against the tick cell-derived bacterial antigens (I/I; homologous antigens) compared to that observed for macrophage culture-grown bacterial antigens (I/D; heterologous antigens) (p values<0.01) (Figure 6).

**Table 5:** Culture and PCR verification of *E. chaffeensis* infection status in dogs infected with either macrophage (DH82) derived or tick cell (ISE6) derived inoculum.

Dog #	Source ofinoculum	Days post infection															Total positives/assessed (% positives)*
		0	2	5	7	9	12	14	16	19	21	29	35	42			
														blood	Liver	Spleen	
6	DH82	-	p	c/p	c	c	p	p	p	p	-	p	p	p	p	p	11/12 (91)*
7	DH82	-	p	c/p	c	c	p	p	-	-	-	p	p	p	p	p	9/12 (75)*
4	ISE6	-	-	-	-	-	-	-	-	-	-	-	-	-	p	p	1/12 (8)**
5	ISE6	-	p	-	p	p	-	-	-	c/p	c	-	c	c	p	p	7/12(58)**
13	Uninfected	-	-	-	-	-	-	-	-	-	-	-	-	-	-	-	0/12 (0)
14	Uninfected	-	-	-	-	-	-	-	-	-	-	-	-	-	-	-	0/12 (0)

The letters c, p or c/p refer to any sample tested positive by culture recovery or PCR or both, respectively. Total average positives in infected animals are 83% and 33% for the DH82 cultured* and ISE6 cultured** inocula, respectively.

## Discussion

Considerable research in understanding the host immune response against *E. chaffeensis* has been carried out using the murine host model and by performing experimental infections with the cultured organisms in canine macrophage cell line (DH82) or in human monocytic cell line (THP1) [21], [23], [39], [40]. *E. chaffeensis*, however, is a tick-transmitted pathogen and causes mostly persistent infections in vertebrate hosts [2], . The murine host is used extensively in mapping immunological responses [8], [17], [21], [23]. However, it is not clear if the outcomes from mouse studies simulate those occurring in hosts acquiring *E. chaffeensis* infections naturally from a tick bite. In particular, the murine host clears the pathogen within a short time period of 14 days, particularly when originating from monocytes/macrophages [15], [18]–[20], [24]. Earlier, we presented evidence that *E. chaffeensis* originating from ISE6 cells induces immune response in mice that is distinct from the immune response developed for the pathogen cultured in DH82 cells [20]. We reported that mice infected with ISE6 cell-derived *E. chaffeensis* exhibited a delay in clearance by about two weeks and the cytokine and B-cell responses were also distinct for this inoculum compared to DH82-derived inoculum [20]. The current study was focused on evaluating infections in the reservoir host by i.v. inoculation with ISE6 and DH82 culture-derived organisms, as well as by tick transmission. Infection assessment was also carried out in dogs (an incidental host) using the i.v. method with cultures grown in ISE6 and DH82 cells. We demonstrated many differences in the *E. chaffeensis* infection progress in animals influenced by the origin of the organism in tick cells or macrophages. We identified greater similarities in the infection progression in deer when inoculum originated from ISE6 cells and by tick transmission. *E. chaffeensis* infection in both the reservoir host (deer) and in the incidental host (dog) resulted in decreased rickettsemia when the inoculum was originated from ISE6 cells.

All *E. chaffeensis* infected animals (deer and dogs) exhibited mild fever. The fever was independent of infections by i.v. inoculation or by tick transmission. Infections in dogs resulted in mild leukopenia, while leukocytosis was observed in infected deer. Persistent fever and leukopenia are two of the most common clinical sings in HME patients [6], [44]–[46] and also observed in the incidental host (dog) in the current study. It is not clear why infection caused leukocytosis in deer. However, as neutrophils and monocytes are the primary blood cells which respond to an infection, it is not uncommon for a bacterial infection to induce leukocytosis [47]. This is the first study reporting clinical signs in both reservoir and incidental hosts which are contrary to the previous reports where no clinical signs were documented [11], [48]–[50]. It is not clear why previous studies could not detect clinical signs; we speculate that the differences may be due to variations in the experimental design and the age of the animals used in each study. For example, infection studies performed by Davidson *et al.* [11] used *E. chaffeensis* isolate recovered from an infected white-tailed deer. It is unclear how the body temperature and total leukocyte counts are assessed in the previous reports. In the current study, we compared the values of infected animals with those of uninfected animals and the infected group animals prior to infections. Fever and thrombocytopenia observed in dogs in the current study is very similar to the observations reported earlier for both natural and experimental infections [41], [51]. Blood smear examinations of samples from infected deer and dogs revealed no detectible *E. chaffeensis* inclusions, suggesting that the rickettsemia levels are significantly low to be detected by microscopy. This is consistent with the earlier studies reporting that *E. chaffeensis* morulae are rarely observed in naturally or experimentally infected deer or dogs [41], [52].

Infection assessment in deer and dogs over a period of several weeks, assessed by more sensitive PCR assay and culture recovery methods, revealed the presence of persisting viable organisms in blood. The organism was detected more frequently, about 80–83% of the time, when infection inoculum originated from macrophage cultures in both deer and dogs. The tick cell-derived inoculum also caused persistence; however, the blood positives detected were considerably lower for this inoculum in both deer and dogs (28–33% of the time). Similar to tick cell cultured inocula infected animals, lower pathogen detection in blood (27% of the time) was also observed in deer acquiring infection by tick transmission. Consistent with these observations, the infection acquisition rates were lower for ticks fed on deer receiving i.v. infection with tick cell cultured inoculum (28%) or by tick transmission (8%) and higher for macrophage cultured inoculum infected animals (78%). The tick acquisition experiment on tick transmitted animals differed two ways compared to those carried out on i.v. infected deer; 1) adult ticks were used for acquisition feeding and 2) ticks on these animals were allowed to feed on the day 56 following transmission feeding of ticks, while ticks on both groups of i.v. infected animals were allowed to feed on day 5 post infection. The possibility that these differences can account for the lowest tick infection acquisition rate for ticks fed on tick transmitted animals cannot be ruled out. Variations in tick infection rates in deer receiving tick cell or macrophage inoculum and tick transmission may have been the reflection of altered rickettsemia in infected deer. In particular, rickettsemia remained consistently low for ISE6 culture-derived i.v. infected animals and tick transmitted deer, whereas consistently high in deer receiving i.v infection from DH82 cultured bacteria. The low infection rates in ticks acquisition fed on tick transmitted deer are very similar to natural infection prevalence rates reported in the literature; *E. chaffeensis* infection prevalence ranged from 2.6% to 7.3% in field collected ticks from various geographical locations [53]–[56]. Despite several studies describing the *E. chaffeensis* infection prevalence in ticks, to date there are no reports documented the presence of inclusions by microscopy. In the current study, we were able to identify *E. chaffeensis* inclusions in a PCR positive tick. This is the first report demonstrating inclusions in a tick. The morula and the *Ehrlichia* inclusions observed in the infected tick midgut epithelial tissue resembled very similar to those observed in infected ISE6 tick cells.

Our current study demonstrates that the infection inoculum originating from macrophage and tick cell environments impact the rickettsemia levels and variations in the IgG responses. The large differences observed and the use of a repeated measures ANOVA analysis of the data to maximize statistical power allowed detection of significant differences despite small numbers of animals. Our research team and others previously reported that the replication within macrophage and tick cell environments cause significant changes in gene expression of *E. chaffeensis* [25]–[28]. The results reported in the current study suggest that the altered gene expression influences how the pathogen progresses in a vertebrate host. In particular, our data suggest that the pathogen gene expression specific to tick cell environment causes a significant reduction in rickettsemia in both reservoir and incidental hosts, which also impacts the infection acquisition by ticks. The origins of infection inocula also influenced the IgG responses. The data support that the IgG responses observed in infected animals were specific to macrophage- and tick cell-derived *E. chaffeensis* antigens. *E. chaffeensis*-specific IgG antibody expression steadily increased and persisted in all infected animals. Interestingly, the IgG made in infected animals was consistently higher for homologous antigens; macrophage culture inoculum infected animals had higher IgG response for the *E. chaffeensis* antigens originating from macrophage cultures (homologous) compared to that observed for tick cell-grown *E. chaffeensis* antigens (heterologous). Similarly, IgG response in i.v. infected animals with tick cell-derived *E. chaffeensis* against tick cell-derived *Ehrlichia* antigens (homologous) is higher, relative to that observed for macrophage culture-derived antigens (heterologous). IgG response in deer receiving infection by tick transmission (the natural mode of transmission) is similar to that observed in animals receiving tick cell-derived i.v. infection; these animals exhibited higher antibody response for tick cell derived *E. chaffeensis* antigens compared to that for macrophage-derived pathogen antigens. These data demonstrate that the tick cell-derived *E. chaffeensis* inoculum is more similar to tick transmission. The inocula differences caused variations in the pathogen’s persistence, tick infection acquisition rates and antigen-specific antibody responses. The host responses in deer receiving tick cell-derived *E. chaffeensis* inoculum more closely resembled the infection by tick transmission.

Infection progression in an incidental host against *E. chaffeensis* was also similar to that observed in deer, when the infections were assessed with the pathogen cultured in macrophages and tick cells. The pathogen persistence in dog and deer was similar; a frequency of pathogen detection was nearly three times higher (83%) for macrophage culture-derived inoculum compared to that for dogs infected with tick cell-derived inoculum (29%). Similarly, the IgG response was greater for the macrophage culture-derived antigens in dogs infected with macrophage inoculum compared to that for tick cell derived antigens. Likewise, dogs infected with tick cell inoculum had significantly higher IgG response against the homologous antigens than that observed heterologous antigens. These results demonstrate that the pathogen infection progression in the reservoir host and in an incidental host is impacted primarily by its prior growth in tick cell or macrophage environments. These results, while similar in many ways to the infection progression in the murine host [20], do reveal unique differences. For example, tick cell cultured *E. chaffeensis* inoculum results in only a short delay in its clearance in the murine host, while it causes persistent infection in deer and dogs. The pathogen persistence was confirmed in liver and spleen samples assessed in deer and dogs at the end points of the study. Further, histopathological changes were observed in lung, liver and spleen of dogs infected with *E. chaffeensis* (our unpublished results; manuscript in preparation).

In mice, the Th1 cytokines and transcriptional activation of a number of Th1-associated genes are upregulated in blood within 10 hours post *E. chaffeensis* infection with organisms originating from macrophages and tick cells [20]. It remains to be seen if similarly the pathogen in the reservoir and incidental hosts causes an increase in the expression of Th1 cytokines. Likewise, the impact of the inoculum originating from tick cell and macrophage environment on the T-cell responses remains to be determined. Future studies focusing on defining the immunological components involved in causing the hosts’ inability to clear the pathogen in both reservoir and incidental hosts will be important in determining how the pathogen evades host responses. The present study, reporting the persistent infections in deer and dogs, is similar to the observations documented in the literature in vertebrate hosts naturally acquiring infections from a tick bite [13], [49]. The current study is the critical first step in gaining insights about how tick-borne *Ehrlichia* species evade host responses, particularly influenced by the distinct antigenic makeup of the bacteria impacted by its growth in macrophage and tick cell environments. The differential protein expression reported previously [25]–[28] may be one of the important mechanisms used by *E. chaffeensis* in support of establishing persistent infections in vertebrate hosts. Investigations focused on understanding the host response against *Ehrlichia* have been mostly carried out in mice or *in* *vitro* with *E. chaffeensis* and the related *Ehrlichia* species cultured in DH82 or THP1 cells [8], [21]–[23], [40]. We did note some host specific differences in the IgG responses in deer and dogs. For example, dogs produce low level IgG to *Ehrlichia* -infected DH82 cells compared to the infected tick cells, which is different compared to the response observed in deer for similar inocula. We believe that these differences may account for host specific immune response variations. Specifically, the responses in the natural host and an incidental host may be inherent to host species or may represent the pathogen’s unique host adaptation strategies. This hypothesis remains to be tested.

The current study suggests that tick cell-derived inoculum or tick transmission would be a better source of inoculum and the experiments should also be carried out in dog or deer to assess the true host immune responses against *E. chaffeensis*. It is evident that much more remains to be understood about the host responses in order to devise methods to control *E. chaffeensis* and other related rickettsial infections in people and other vertebrate animals.
